# Identification of Key Drought Stress-Related Genes in the Hyacinth Bean

**DOI:** 10.1371/journal.pone.0058108

**Published:** 2013-03-05

**Authors:** Lu-Ming Yao, Biao Wang, Lin-Jing Cheng, Tian-Long Wu

**Affiliations:** Plant Science Department, School of Agriculture and Biology, Shanghai Jiao Tong University, Shanghai, China; National Taiwan University, Taiwan

## Abstract

Hyacinth bean (*Lablab purpureus* [Linn.] Sweet) possesses excellent characteristics for field production, but the response of this plant to drought stress has not been described at the molecular level. Suppression subtraction hybridization (SSH) is an effective way to exploit key factors for plant responses to drought stress that are involved in transcriptional and metabolic activities. In this study, forward and reverse SSH libraries were generated from root tissues of the drought-tolerant hyacinth bean genotype MEIDOU 2012 under water–stress conditions. A total of 1,287 unigenes (94 contigs and 1,193 singletons) were derived from sequence alignment and cluster assembly of 1400 ESTs, and 80.6% of those hit against NCBI non-redundant (nr) database with *E value* <1E−06. BLASTX analysis revealed that the majority top matches were proteins form *Glycine max* (L.) Merrill. (61.5%). According to a gene ontology (GO) functional classification, 816 functionally annotated unigenes were assigned to the biological process category (74.1%), and 83.9% of them classified into molecular function and 69.2% involved in cellular component. A total of 168 sequences were further annotated with 207 Enzyme Commission (EC) codes and mapped to 83 different KEGG pathways. Seventeen functionally relevant genes were found to be overrepresented under drought stress using enrichment analysis. Differential expression of unigenes were confirmed by quantitative real-time PCR assays, and their transcript profiles generally divided into three patterns, depending on the expression peaked levels after 6, 8 or 10 days dehydration, which indicated that these genes are functionally associated in the drought-stress response.

## Introduction

The hyacinth bean (*Lablab purpureus* [Linn.] Sweet), with a large biomass and strong capacity for nitrogen fixation, is widely cultivated in China, Southeast Asia, India, Australia and eastern areas of Africa [1]. Hyacinth bean possesses excellent characteristics for field production, such as tolerance to drought and salinity stress [1], [2]. The responses of plants to drought stress are complex, including the interaction between the environment and various metabolic pathways [3]. Plant responses to drought stress have been described at both the physiological and molecular levels, including the profiling of alterations in transcripts, protein and metabolites. However, the response of hyacinth bean to drought stress has not been described at the molecular level, except for general information concerning the biochemical and physiological responses of this plant to water deficiency and other abiotic stresses [4].

Drought has a significant effect on each plant tissues. Root is the first plant organ to be negatively impacted by soil water deficit, and usually used to investigate about diverse responses to dehydration. Molecular approaches have been demonstrated one major quantitative trait locus (QTL) that accounts for 33% of the variation in root biomass, which is one of the principal index that confer a drought-tolerance advantage to plants, among many other constructive mechanism responses to water deficit [5]. Many plants, including loblolly pine (*Pinus taeda*) and chickpea (*Cicer arietinum*) have identified candidate genes for drought tolerant by transcriptional analysis of root tissues [6], [7].

Suppression subtractive hybridization (SSH) is a widely used method for separating DNA molecules that distinguishes two closely related DNA samples. This technique is very useful for studies involving plant development and the abiotic/biotic response [8], [9], [10]. SSH has been employed to identify and characterize differentially expressed genes under drought stress, leading to the discovery of a number of proteins related to the biotic/abiotic stress response, as well as transcription factors, such as *SiDREB2* in foxtail millet (*Setaria italica*) [11], *OsWR1* in rice (*Oryza Sativa*) [12] and *StMYB1R-1* in potato (*Solanum tuberosum*) [13],and all of them involve in the drought tolerance enhancement, and *CBF* genes [14] in Arabidopsis and *SiNAC* from foxtail millet [15] and these transcripts mediate plants responses to abiotic stresses. SSH has become an effective way to exploit genes potentially involved in plant responses to drought stress, especially factors associated with transcriptional and metabolic activities. However, it has not yet been reported to use for differentially expressed transcripts (both up- and down-regulated) identification in hyacinth bean under drought stress at the seedling stage of the plant.

In this study, we constructed forward and reverse SSH libraries using the root tissues of drought-tolerant hyacinth bean genotype MEIDOU 2012, specifically focusing on seedlings under drought stress. Information concerning the genome of hyacinth bean and its molecular mechanisms of abiotic stress tolerance is scarce compared with that of other leguminous crops, such as *Glycine max* and *Medicago sativa*. EST sets related to up- and down-regulated genes were generated, and the expression patterns of these genes were examined in plants subjected to water stress for 2, 4, 6, 8 or 10 days. Our results enabled us to identify key novel genes associated with drought stress in hyacinth bean.

## Materials and Methods

### Plant materials

Hyacinth bean genotype MEIDOU 2012 with large and prolific root system was conducted in a climate-controlled chamber at 28°C, with an illumination intensity of 200 µmol m^−2^ s^−1^ and relative humidity of 80%, under a 16 h/8 h light-dark cycle. Seeds were sowed in soil in PVC cylinders of 10-cm diameter and 9-cm height. Water stress (WS) treatment was applied at10 days after germination (DAG) by withholding watering for 10 days, while the control group was well watered (WW) every day. Root tissues from WW and WS plants were harvested separately every other day, from 10–20 DAG, frozen in liquid nitrogen and stored at −80°C for RNA extraction.

### RNA Isolation

For each sample, 0.1 g of root tissue was quickly ground into a fine powder in liquid nitrogen with a mortar, and total RNA was isolated using UNlQ-10 Total RNA Isolation Kit (Sangon, Shanghai China). To construct the SSH libraries, equal amounts of total RNA (100 µg from each replicate) from four replicates of WW and WS were pooled separately, and mRNA was isolated using a Poly (A) mRNA Purification Kit (Sangon, Shanghai China) from total RNA samples.

### SSH Library Construction

**Figure 1:**
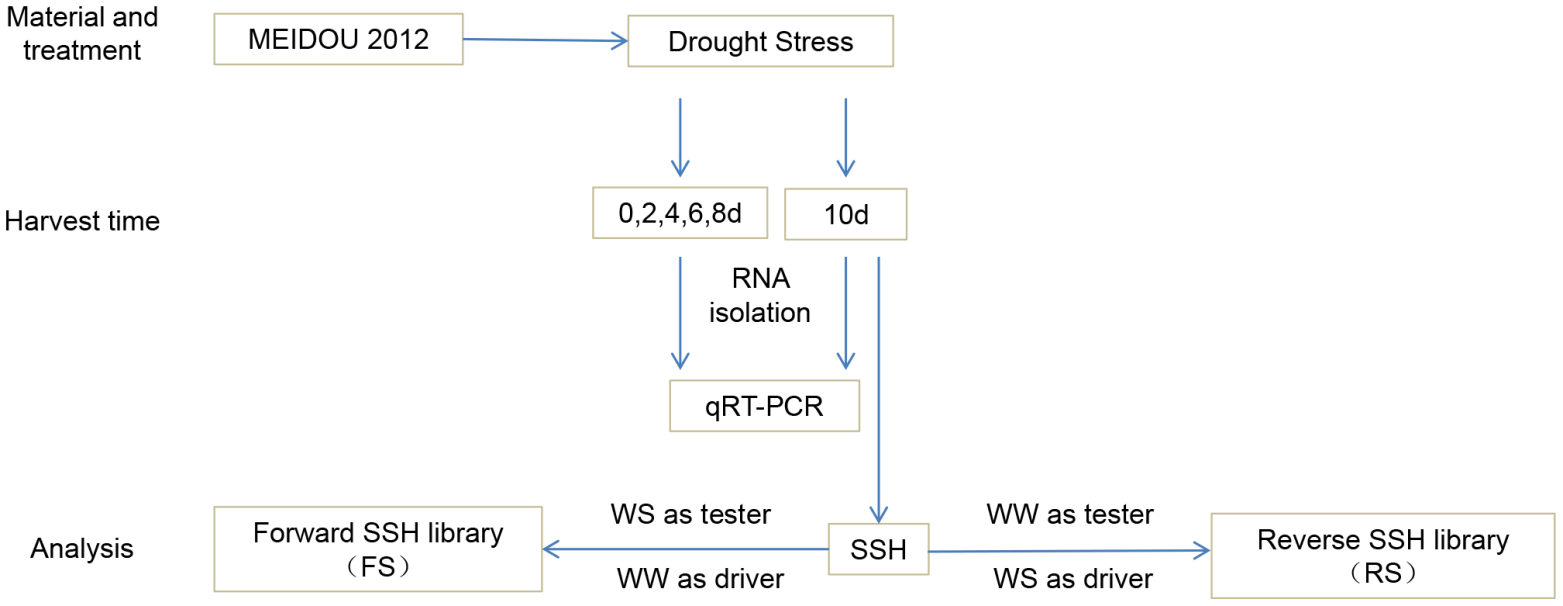
Flow chart of material harvest and data analysis. *Lablab purpureus*, drought-tolerant genotype MEIDOU 2012, was used for cDNA SSH library construction. Both forward (FS) and reverse subtractions (RS) were generated using reciprocal samples, and expression analysis was performed during different periods of drought stress.

A forward-subtracted library (FS) was constructed with cDNA from WS (tester) and WW (driver) to isolate up-regulated genes in roots under drought stress, and a reverse-subtracted library (RS) was constructed to isolate down-regulated genes in drought stress (Figure 1).

SSH libraries were constructed using a PCR-Select™ cDNA Subtraction Kit (Clontech, US), starting with 2 µg of mRNA from the tester and driver samples. Subtraction was performed, according to manufacturer’s instructions, to identify the transcript enriched in one sample relative to the other. Subtracted cDNAs were ligated into a pBluescript II SK(−) vector and transformed into *E. coli* strain DH10B (Invitrogen, Carlsbad, CA, USA).

Positive clones from LB plates containing 50 mg/L ampicillin were selected for PCR amplification to identify the insert sizes.

### Differential Screening with Reverse Northern Blotting

To screen differentially expressed EST from libraries, equal amounts of purified PCR-amplified products (100 ng) were spotted onto nylon membranes (PALL, US) in a 96-well format. Each blot was prepared in triplicate. A PCR-amplified product of actin was used as a housekeeping gene for normalization of the signals between the blots, and phosphinothricin N-acetyltransferase (*bar*) was used as a negative control to correct for background noise. Samples were spotted onto the membranes and cross-linked by baking the membranes at 120°C for 30 min.

The cDNA was synthesized from equal amounts of mRNA (100 ng) from WW and WS using a PrimeScript RT Reagent Kit (Takara, Japan). DIG-labeled cDNA synthesis was performed using a DIG High Prime DNA Labeling and Detection Starter Kit II (Roche, Germany). The products were used as probes for hybridization with the prepared nylon membranes, according to the manufacturer’s instructions.

### Sequence Processing

All sequences were checked with SeqTrim [16] (http://www.scbi.uma.es/cgi-bin/seqtrim/seqtrim_login.cgi). Adaptors, poly A/T ends, pBluescript II SK(-) vector sequences and sequences less than 100 bp were trimmed. A total of 5% of sequences selected randomly was also processed manually using BLAST [17] to confirm that the processed sequences were none of any adaptors, poly A/T ends, vector sequences and low quality sequences. High quality sequences were assembled by SeqMan^TM^7.1.0 (DNASTAR, Lasergene) [18], requiring sequences with at least 95% sequence identity and a minimum overlap of 40 bp. All contigs and singletons were combined into the differentially expressed unigene data sets.

### Sequence Analysis

Similarity analysis of sequences in the unigene data sets by the BLASTX of NCBI BLAST program [18] (parameters: *E-value* 1.0E−6, HSP length cutoff 33, Blast Hits 20) was performed against the NCBI non-redundant (nr) database.

BLAST2GO (version 2.5.0) [19] was used for mapping and annotation (parameters: E-Value-Hit-Filter 1.0E−6, Annotation Cutoff 55, gene ontology [GO] Weight 5, Hsp-Hit Coverage Cutoff 20). The genes were characterized using GO terms, i.e., molecular function, biological process and cellular component. Enzyme mapping of annotated sequences was performed using direct GO to Enzyme mapping and were used to query the Kyoto Encyclopedia of Genes and Genomes (KEGG) to define the KEGG orthologs (KOs). These KOs were then plotted into whole metabolic pathways using the KEGG mapping tool.

GO enrichment analysis was performed with the Fisher exact test using the GOSSIP module [20] integrated in the Blast2GO package. For GO enrichment analysis, all GO terms with a cutoff threshold of p-value≤0.05 were considered to be differentially enriched between the two sets of libraries.

### Real-time Quantitative RT-PCR

Differentially expression analysis of the genes was performed with real-time quantitative RT-PCR in triplicate using cDNA synthesized from 5 µg total RNA of WW and WS separately using a PrimeScript RT Reagent Kit (Takara, Japan). Actin was used as an internal control for normalization of variations in cDNA samples. PCR primers were designed using Primer3 (http://www.ncbi.nlm.nih.gov/tools/primer-blast/), with the parameters of optimum primer GC content of 50%, primer Tm = 55–65°C, primer length 20–25 nucleotides and an expected amplicon size of 80–200 bp. Real-time quantitative RT-PCR was performed using SYBR Premix Ex TaqTM II (Takara, Japan), and the PCR program was set as 30 s at 95°C, 40 cycles each of 30 s at 94°C, 10 s at 55°C and 30 s at 72°C. PCR amplification and data collection were performed using an FTC 3000 (FungLyn Biotech, China) (Figure 1). To minimize experimental error, three independent cDNA replicates for each sample were used.

## Results

### SSH Library Construction and Screening

**Figure 2:**
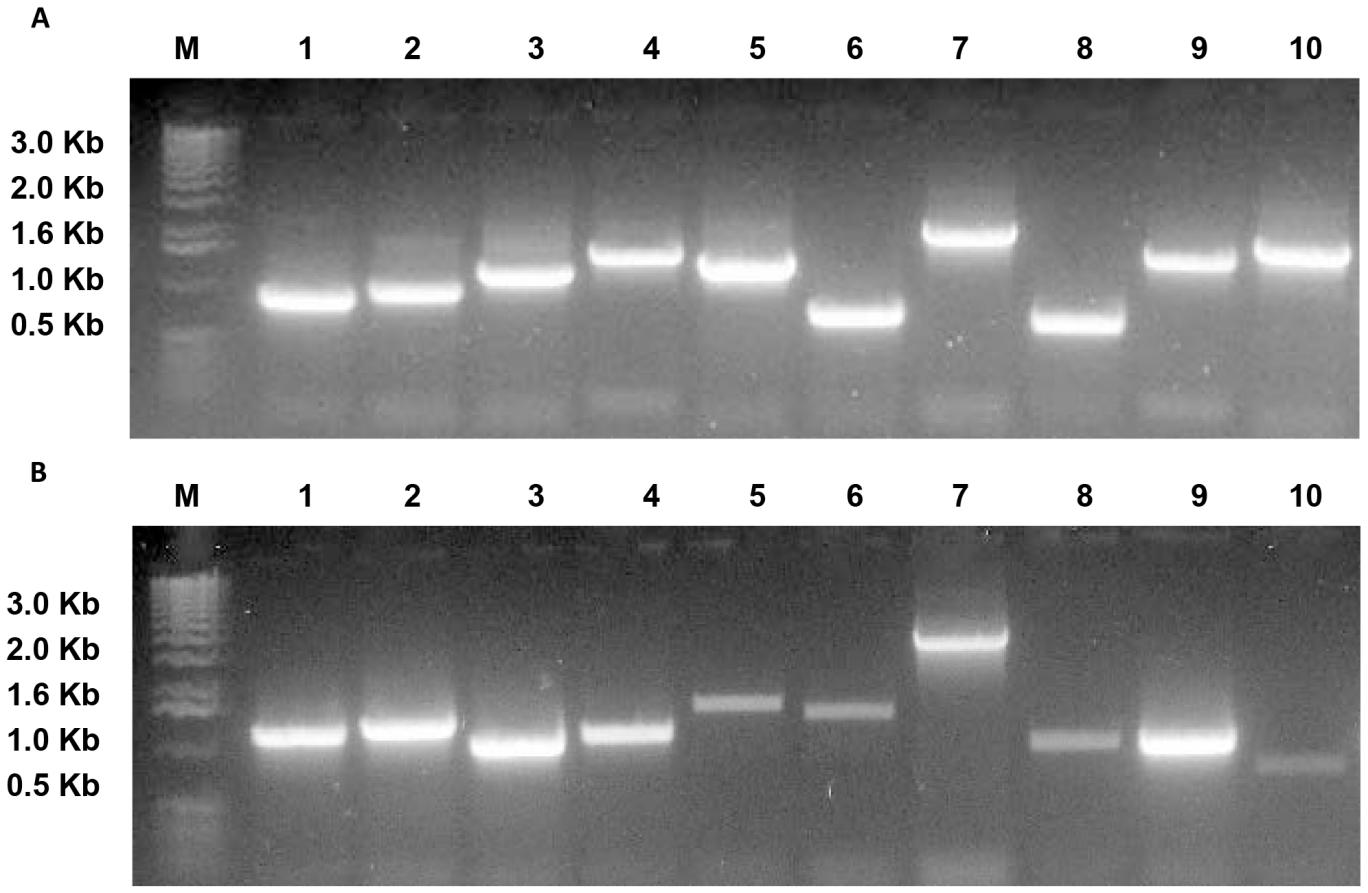
Insert sizes forward and reverse SSH library. The insert sizes were 0.5–2.0 kb, with an average size of 1.2 kb in forward SSH library (A) and 1.0 Kb in reverse SSH library (B). M: markers; Lanes 1–10: insert sizes of 10 clones from forward SSH library (A) and reverse SSH library (B).

Samples from WW and WS that were under drought stress for 10 days were used to construct forward and reverse subtracted libraries to identify differentially expressed genes in the roots of hyacinth bean seedlings. The insert sizes of clones in the library were 0.5–2.0 kb, with an average size of 1.2 kb in FS library and 1.0 Kb in RS library (Figure 2). The total number of clones in FS and RS were 2.03×10^5^ and 2.1×10^5^, respectively.

**Figure 3:**
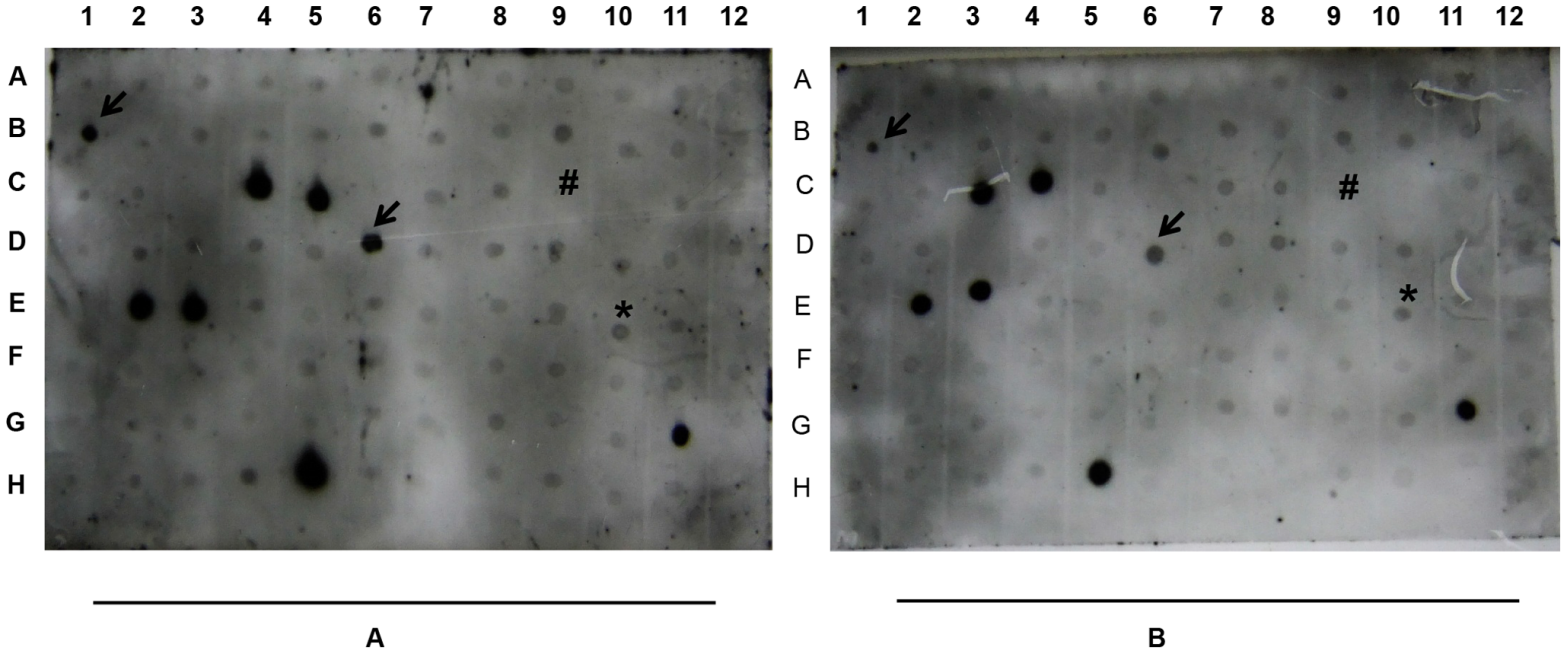
Microarray hybridization of SSH cDNA clones. Nylon membrane containing cDNA spots from a library generated from FS and RS hybridized with DIG-labeled cDNA probes synthesized from WS (A) and WW (B) plants. The blots indicated with arrows were selected for further analysis. Actin (*) was used as an internal control to normalize the signals from two different blots, and Bar (#) was used as a negative control to enable the subtraction of background noise.

Two libraries were screened by reverse northern blotting (Figure 3), and a total of 1,525 clones (774 clones in FS and 751 clones in RS) out of 2,899 clones (1,499 clones in FS and 1,400 clones in RS) displayed at least a 1.5-fold level of induction were selected for further analysis.

### EST Assembly

A total of 1,525 clones which displayed at least a 1.5-fold level of induction in libraries were selected for sequencing and EST assembly. A total of 1,400 high quality sequences (723 sequences in FS and 677 sequences in RS), with an average length of 864 bp, were generated after trimming adaptors, poly A/T ends, pBluescript II SK(−) vector sequences and sequences less than 100 bp. With EST assembly, 1,287 unigenes (638 unigenes in FS and 649 unigenes in RS) containing 94 contigs and 1,193 singletons were obtained and developed as FS and RS unigene sets (Table 1). Each contig had 2–15 ESTs, with an average length of 811 bp, and 64.3% of the contigs contained two ESTs, while 19.1% contained three ESTs. All EST sequences have been deposited in the dbEST division of GenBank (accession: JZ150029–JZ151480).

**Table 1:** Summary of drought responsive SSH libraries.

Name of SSH library	Tester	Driver	No. of clones	High quality sequences	No. of contigs	No. of singletons	No. of Unigenes	Description of transcript clones
FS	WS root	WW root	774	723	68	570	638	Up-regulated in root tissues
RS	WW root	WS root	751	677	26	623	649	Down-regulated in root tissues
Total			1525	1400	94	1193	1287	Differentially regulated in root tissues

FS: Forward subtraction, RS: Reverse subtraction, WW: Well watered, WS: Water stressed plants.

### Annotation and Functional Cataloging

**Figure 4:**
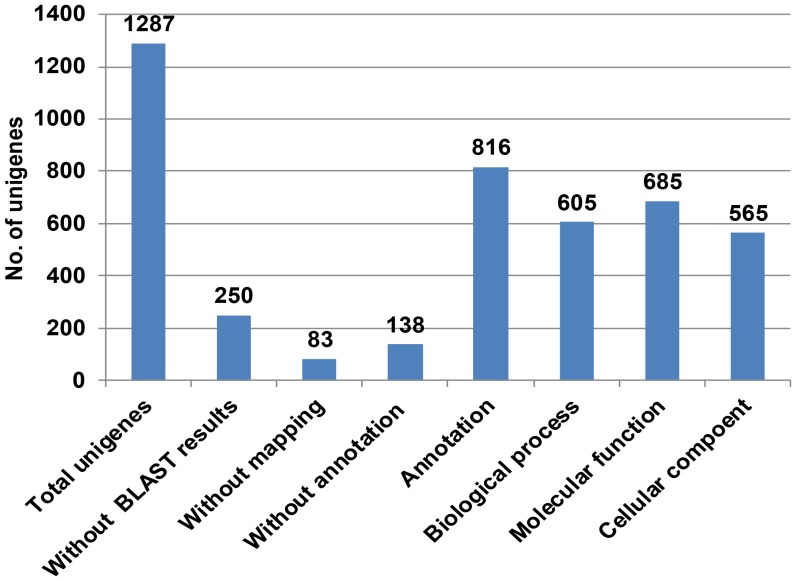
Annotation statistics of unigenes. Unigenes annotated as known proteins with E-value threshold 1.0E−6. Numbers of total unigenes, without BLAST results, without mapping and without annotation, are presented. And the number of unigenes annotated with GO and grouped in 3 major categories, biological progress, molecular function and cellular component.

**Figure 5:**
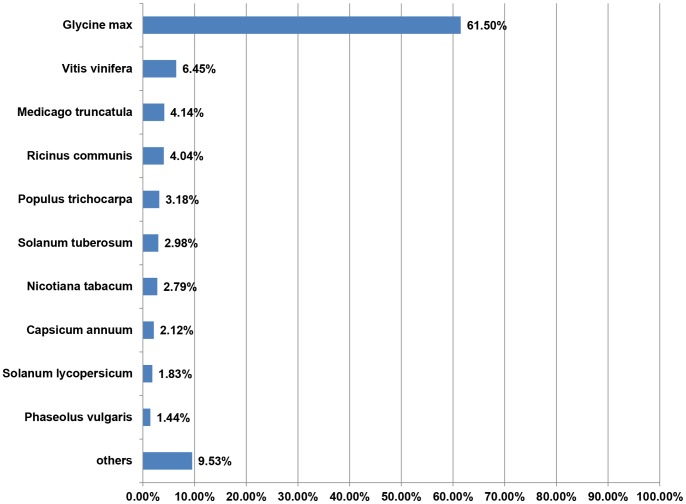
Distribution of similarity between *Lablab purpureus* unigene data sets and other species. Unigenes were grouped according to BLAST top-hits species, as determined by protein similarity search BLASTX *E-values* <1E−06.

Similarity analysis of 1,287 sequences in the unigene sets were analyzed by BLASTX against the NCBI non-redundant (nr) database. The results showed that 80.6% of the sequences in the data sets hit against NCBI non-redundant (nr) database with *E value* <1E−06 (Figure 4). BLASTX analysis revealed that the majority top matches were proteins form *Glycine max* (61.50%, 639 unigenes), followed by *Vitis vinifera* (6.45%, 67 unigenes), and the similarity between hyacinth bean and *Medicago truncatula* or *Phaseolus vulgaris*, which are all belong to legume species, are 4.14% (43 unigenes) and 1.44% (15 unigenes) respectively (Figure 5).

**Figure 6:**
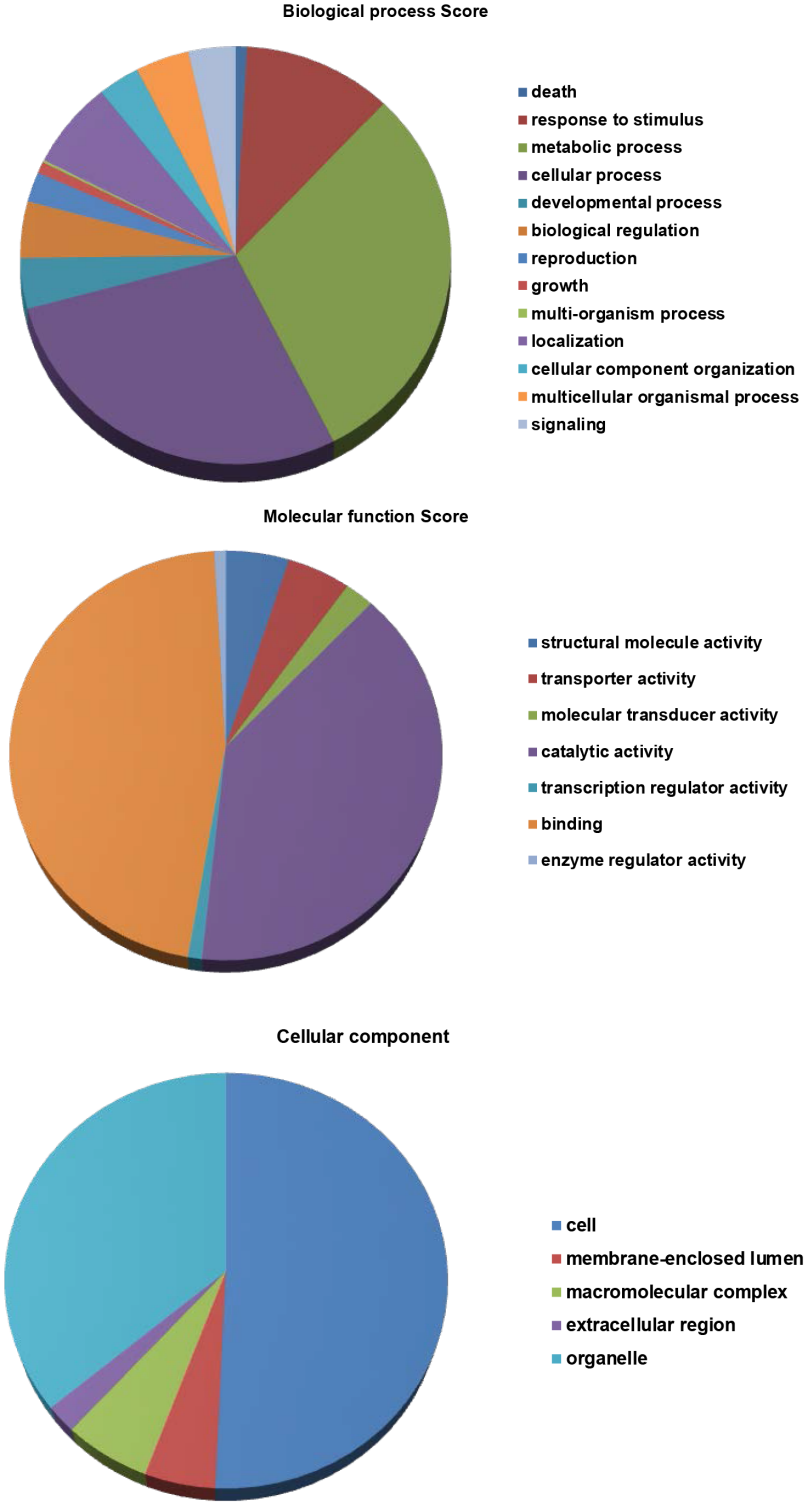
Functional classification of differentially expressed unigenes according to BLAST2GO. The GO classifications of *L. purpureus* unigenes, including biological process, molecular function and cellular component.

Mapping and functional annotation of unigenes by Blast2GO resulted in GO functional classification terms for 1,037 (80.6%) sequences in the data sets, of which 816 (78.7%) unigenes were functionally annotated (GO consensus) and 138 (13.3%) sequences were mapped but not annotated (Figure 4). At the second level GO analysis, 605 out of 816 unigenes functionally annotated (74.1%) were assigned to the biological process category, 685 sequences (83.9%) classified into the molecular function category and 565 sequences (69.2%) sorted out the cellular component category (Figure 4). The biological process category “metabolic process” was the most prevalent (30.4% of sequences), followed by “cellular process” (28.4%) and “response to stimulus” (11.2%), while in the molecular function category, “binding” was the most prevalent (46.4%), followed by “catalytic activity” (39.5%), “structural molecule activity” (4.95%) and “transporter activity” (4.95%). In the cellular component category, “cell” was the most dominant term, followed by “Organelle” (36.1%) and “macromolecular complex” (6.1%) (Figure 6).

Of the 816 annotated unigenes, 168 sequences (87 sequences in FS and 81 sequences in RS) were annotated with 207 Enzyme Commission (EC) codes (111 EC codes in FS and 96 EC codes in RS) and mapped to 83 KEGG pathways (68 pathways in FS and 61 pathways in RS). KEGG metabolic pathways that were well-represented by unigenes included amino acid metabolism (31 enzymes), alkaloidal biosynthesis (4 enzymes), starch and sucrose metabolism (11 enzymes), nitrogen metabolism (11 enzymes) and flavonoid biosynthesis (5 enzymes).

**Figure 7:**
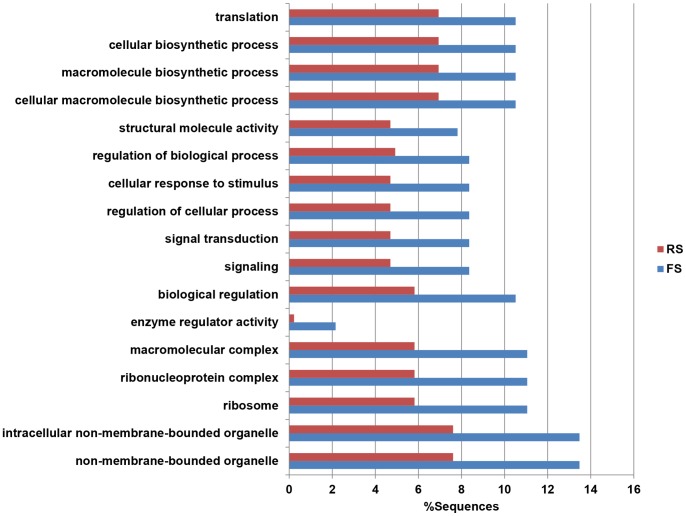
Differential GO terms between ESTs derived from forward and reversr libraries under drought stress. GO enrichment analysis of ESTs generated from forward SSH and reverse SSH libraries using Fisher’s exact test with a p-value mode cutoff of p≤0.05. The number of transcripts associated with a specific GO term is represented as the percentage of functionally annotated ESTs in a library.

Identification of overrepresented and underrepresented GO terms from a given list of genes from different libraries may help elucidate the functional relevance of these genes under drought stress. A total of 17 GO terms that were differentially represented between FS and RS were identified by GO enrichment analysis, and all 17 terms were overrepresented in FS (Figure 7). Several overrepresented terms were associated with important pathway in plants under drought stress, such as phenylalanine metabolism, flavonoid biosynthesis and proline metabolism.

### Confirmation of SSH Data by Real-time Quantitative RT-PCR

**Figure 8:**
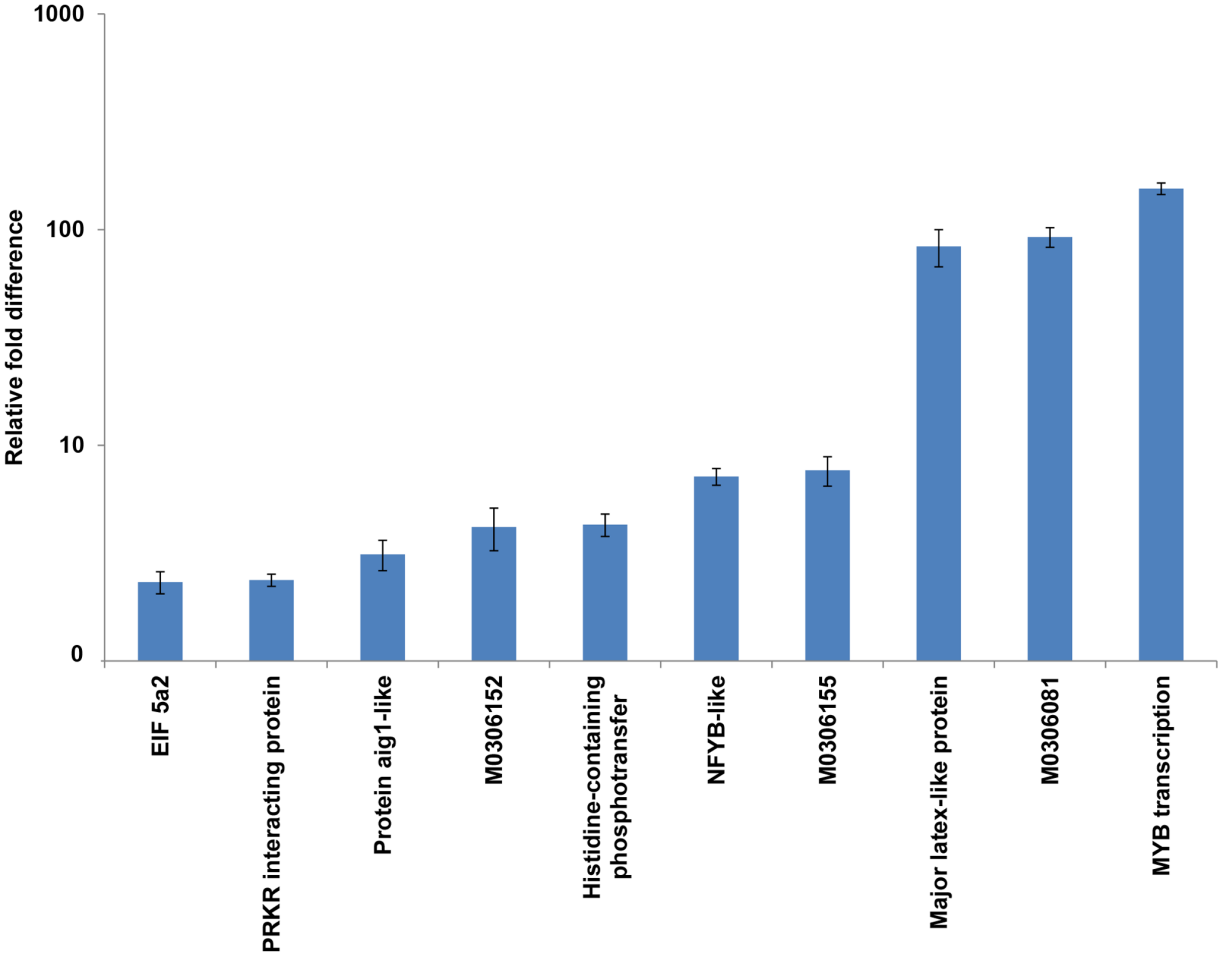
Expression analysis of selected unigenes with altered expression in response to 10 days of drought stress. Relative expression levels (fold difference) of ten selected unigenes altered in plants exposed to drought stress for 10 days were evaluated using qPCR analysis. Error bars represent standard error of the mean (Number of replication n = 3). Unigenes used for qPCR analysis were EIF 5a2, PRKR interacting protein, Protein aig1-like, histidine-containing phosphotransfer, NFYB-like, major latex-like protein, MYB transcription and M0306152, M0306081, M0306155 with no BLAST results.

To validate the results of SSH data, 10 differentially expressed unigenes were analyzed by quantitative real-time PCR, including three novel genes (M0306155, M0306152 and M0306081) and seven genes that putatively encode protein aig1-like (JZ150379), PRKR interacting protein (JZ150202), NFYB-like (JZ150176), MYB transcription (JZ150356), major latex-like protein (JZ150158), histidine-containing phosphotransfer (JZ150231) and EIF 5a2 (JZ150235) respectively. All of the unigenes were up-regulated in plants under 10 days of drought stress, with a relative fold difference of 2.32–155; the most differentially expressed genes were genes encoding MYB transcription, M0306081 and major latex-like protein, with changes in expression of 155-, 92- and 84-fold, respectively, followed by M0306155 (7.65-fold), NFYB-like (6.17-fold), histidine-containing phosphotransfer (4.29-fold), M0306152 (4.18-fold), protein aig1-like (3.12-fold), PRKR interacting protein (2.37-fold) and EIF 5a2 (2.32-fold) (Figure 8).

### Identification of Unigenes Expression Pattern

**Figure 9:**
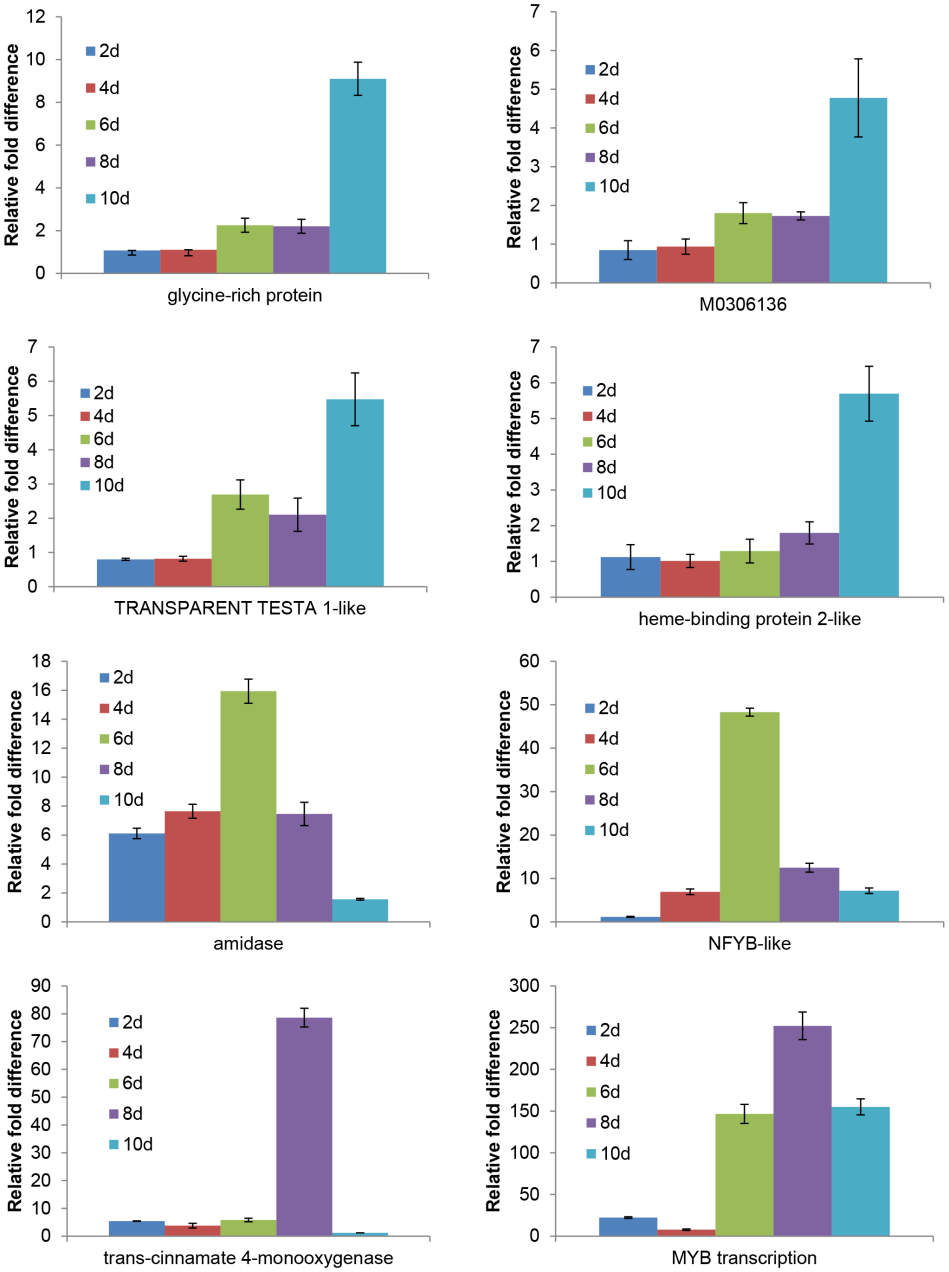
Expression analysis of selected unigenes with altered expression at various times in response to drought stress. Relative expression levels (fold difference) of eight selected unigenes under drought stress for 2, 4, 6, 8 or and 10 days were evaluated using qPCR analysis. Error bars represent standard error of the mean (Number of replication n = 3). Unigenes used for qPCR analysis were glycine-rich protein, TRANSPARENT TESTA 1-like, heme-binding protein 2-like, M0306136 (JZ150181) with no BLAST results, amidase, NFYB-like, trans-cinnamate 4-monooxygenase and MYB transcription.

Differential expression of unigenes in response to drought stress on day 2, 4, 6, 8 and 10 were analyzed by Quantitative real-time PCR. The data showed that the expression profiles can generally be divided into three patterns (Figure 9). The induction of most unigenes continued to increase until the end of the experiment, including putative genes assigned to cellular components category in GO analysis, such as genes encoding glycine-rich protein (JZ150166), transparent testa 1-like (JZ150196) and heme-binding protein 2-like (JZ150210). The transcripts of some genes reached their maximum induction levels 6 days after withholding water, and among these, a putative nuclear transcription factor, NFYB-like, was detected, along with genes encoding proteins with catalytic activity, such as amidase (JZ150184). The expression of other genes reached a maximum transcription level on day 8 of water stress treatment, which mainly included genes putatively related to secondary metabolic processes, as well as some putative transcription factors, such as trans-cinnamate 4-monooxygenase (JZ150189) and the MYB transcription factor.

## Discussion

### Drought Stress and Differentially Expressed Genes

Plant growth and productivity are adversely affected by various biotic and abiotic stress factors, while drought is one of the major abiotic stresses that adversely affect crop growth and yield [3]. Recent studies of water deficit have focused on seedlings and terminal drought, i.e., the effects of drought stress on seedlings and flowering/podding. Seedling drought adversely affects plant growth and development. The gene of the drought-inducible glycine-rich RNA-binding protein (GR-RBP) was isolated and characterized to determine its role in the response of apple (*Malus prunifolia*) seedlings to drought stress using leaves from plants under water stress [21], while the overexpression of OsRDCP1, a rice RING domain-containing E3 ubiquitin ligase, was found to increase tolerance to drought stress in rice (*Oryza sativa*) [22]. A novel NAC family gene, *CarNAC1*, was cloned from chickpea (*Cicer arietinum*), a drought-tolerant leguminous crop [23]. Specific and non-specific biochemical and physiological responses of hyacinth bean to drought stress have been reported [4]. Peroxidase (POX) and glutathione reductase (GR) increased when catalase (CAT) decreased in hyacinth bean leaves under drought stress, and same results have been reported in chickpea subjected to salt stress [24]. Moreover, a new isozyme of POX was observed in roots of stressed hyacinth bean [4]. The labyrinth of molecular mechanism under water deficit in hyacinth bean remained yet.

In the present study, we analyzed the response of hyacinth bean seedlings to drought stress and examined metabolic pathways related to this process using subtracted libraries of root tissues. We found that genes related to pathways previously reported to be involved in the drought stress response were altered upon exposure to drought-stress conditions, including genes associated with amino acid metabolism, alkaloidal biosynthesis and flavonoid biosynthesis.

Water deficit during plant flowering and podding adversely affect plant productivity, and studies of terminal drought have shown that root density and biomass are correlated with the level of drought tolerance of a plant [6], [25]. Comparative analysis of ESTs between drought-tolerant and -susceptible genotypes of chickpea under terminal dehydration revealed that more than 50% of the identified genes were different from datasets generated by water deficit of chickpea seedlings [26], which indicated that the responses of plants to drought stress were diverse in each development stage. We focused on the seedlings of the hyacinth bean response to water deficit and tried to define the key regulated unigenes.

In-air wilting [12], PEG treatment [11], [27], withholding water [28] and dry down [7] are the reported methods used to study plant responses to dehydration. Both in-air wilting and PEG treatment impose artificial conditions on plants, leading to water-stress responses that differ from those found in nature. Furthermore roots exposed to PEG may become oxygen deficient, making this treatment inappropriate for inducing long-term water stress, while in-air wilting treatment induced dehydration severely in hours, making these treatment both inappropriate for inducing long-term water stress. In contrast, dry down, a process that induces gradual and progressive water-deficit stress is not appropriate for inducing drought in seedlings because of the long duration of the treatment. For seedling water-deficit stress, withholding water, simulating natural dehydration and inducing drought for a suitable period, is a favorable method. We imposed water stress by withholding water for 10 days in hyacinth bean seedlings 10 DAG. We therefore employed a desired method for seedling drought studies that simulates the type of stress encountered under field conditions.

### Studies of Plant Responses to Abiotic Stress using SSH Libraries

SSH is a widely used method for understanding gene regulation mechanism, distinguishing novel genes and exploring new functions of genes, and it is efficient and sensitive for plant development and abiotic response research. For example, two novel genes involved in seed development, lipid metabolism and protein metabolism in *Brassica napus* seed development were previously identified [29]. And also two up-regulated genes encoding putative transcription factors were identified during somatic embryogenesis in cucumber (*Cucumis sativus*) [30].

Concerning abiotic-response research, an interaction network of salt-responsive genes was constructed by analyzing the expression profiles of early responsive genes in upland cotton (*Gossypium hirsutum*) exposed to salt stress [31]. Moreover, genes involve in two response processes (defense at the early stage and adaption at the late stage) were identified in the roots of maize (*Zea mays*) seedlings in the late stage of waterlogging [32].

In our study, abundant novel ESTs related to hyacinth bean responses under seedling drought stress were identified, and various metabolic pathways were discovered, including pathways involved in amino acid metabolism, alkaloidal biosynthesis, starch and sucrose metabolism, nitrogen metabolism and flavonoid biosynthesis.

### Possible Genes or Factors Related to Drought Responses and Research Progress

Plant responses under water stress are complex processes that involve broad metabolic and synthetic pathways. Phenylalanine metabolism and flavonoid biosynthesis are important pathways in the plant response to abiotic/biotic stress; the gene encoding phenylalanine ammonia lyase is up-regulated in response to dehydration, salinity stress and wounding [33], [34], [35]. In addition, phenylalanine and tyrosine contents increase during wounding [36]. Flavonoids, which are secondary metabolites, are involved in various types of stress [37], [38], [39], and water deficit stress was found to decrease chlorophyll content while increasing the content of flavonoids [40]. Besides, there still a variety of genes involved in cellular component category were reported as functional roles during stress adaptation. *BhGRP1* encoding a cell wall localized glycine-rich protein play a role in cell wall maintenance and repair during dehydration and rehydration [41]. And TSPO, a heme-binding, cell membrane localized protein was induced be stress, and scavenged porphyrin during stress in plants [42].

In this study, the genes encoding enzymes involved in the phenylalanine metabolism, flavonoid biosynthesis pathways and putative genes encoding protein located on cell membrane were found to be differentially expressed under drought stress, which is in agreement with other reports.
